# Impact of Interleukin-6 –174 G>C Gene Promoter Polymorphism on Neuroblastoma

**DOI:** 10.1371/journal.pone.0076810

**Published:** 2013-10-21

**Authors:** Francesca Totaro, Flora Cimmino, Piero Pignataro, Giovanni Acierno, Marilena De Mariano, Luca Longo, Gian Paolo Tonini, Achille Iolascon, Mario Capasso

**Affiliations:** 1 Dipartimento di Medicina Molecolare e Biotecnologie Mediche, Università degli Studi di Napoli Federico II, Naples, Italy; 2 CEINGE – Biotecnologie Avanzate, Naples, Italy; 3 Terapia Immunologica, IRCCS AOU San Martino-IST-Istituto Nazionale per la ricerca sul cancro, Genoa, Italy; 4 IRCCS AOU San Martino-IST, National Cancer Research Institute, Genoa, Italy; 5 Laboratory of Neuroblastoma, Onco/Hematology Department SDB University of Padua, Pediatric Research Institute, Padua, Italy; Sanjay Gandhi Medical Institute, India

## Abstract

**Background:**

Common variants in DNA may predispose to onset and progression of neuroblastoma (NB). The genotype GG of single nucleotide polymorphism (SNP) rs1800795 (−174 G>C) in interleukin (IL)-6 promoter has been associated with lower survival of high-risk NB.

**Result:**

To evaluate the impact of *IL-6* SNP rs1800795 on disease risk and phenotype, we analyzed 326 Italian NB patients and 511 controls. Moreover, we performed *in silico* and quantitative Real Time (qRT)-PCR analyses to evaluate the influence of the SNP on gene expression in 198 lymphoblastoid cell lines (LCLs) and in 31 NB tumors, respectively. Kaplan-Meier analysis was used to verify the association between *IL-6* gene expression and patient survival. We found that *IL-6* SNP is not involved in susceptibility to NB development. However, our results show that a low frequency of genotype CC is significantly associated with a low overall survival, advanced stage, and high-risk phenotype. The *in silico* (*p* = 2.61×10^−5^) and qRT-PCR (*p* = 0.03) analyses showed similar trend indicating that the CC genotype is correlated with increased level of *IL-6* expression. In report gene assay, we showed that the −174 C variant had a significantly increased transcriptional activity compared with G allele (*p* = 0.0006). Moreover, Kaplan-Meier analysis demonstrated that high levels of *IL-6* are associated with poor outcome in children with NB in two independent gene expression array datasets.

**Conclusions:**

The biological effect of SNP *IL-6*–174 G>C in relation to promotion of cancer progression is consistent with the observed decreased survival time. The present study suggests that SNP *IL-6*–174 G>C may be a useful marker for NB prognosis.

## Introduction

Neuroblastoma (NB) is a neuroendocrine tumor arising from neural crest element of the sympathetic nervous system. NB accounts for more than 7% of malignancies in children and around 15% of pediatric oncologic deaths [1]. In recent years, we have demonstrated that common DNA variants located in diverse genes (*LINC00340*, *BARD1*, *LMO1*, *DUSP12*, *HSD17B12*, *DDX4*, *IL31RA*, *HACE1* and *LIN28B*) predispose to NB onset and progression by genome-wide association studies (GWAS) [2]–[6]. The DNA variants of *LMO1*, *LIN28B* and *BARD1* have also shown relevant biological functions in NB [4], [6]–[7]. Moreover, most of these NB GWAS-risk loci have been recently replicated in an Italian population and when combined have shown the potential to distinguish subgroups of patients at different risks of developing NB [8]. Our observations suggested that NB tumorigenesis could be the result of different genetic alterations, which might also influence the clinical outcome of disease.

Interleukin (IL)-6 is a pro-inflammatory cytokine that plays an active role in neoplasia, bone metabolism and iron homeostasis. Recent studies have demonstrated a role for *IL-6* in progression and development of multiple myeloma, colon cancer, melanoma, renal cancer, Hodgkin's disease, non-Hodgkin's lymphoma, prostate cancer, breast cancer and esophageal cancer [9]–[10].

The *IL-6* expression has been associated to increased expression of adhesion receptors on endothelial cells and of growth factors, suggesting that *IL-6* exerts a stimulation activity on microenvironment promoting the metastasis [11]–[12]. Recent scientific literature suggests that *IL-6* plays a role also in NB tumor progression. Indeed, it has been demonstrated that elevated peripheral blood *IL-6* levels correlates with NB progression and development [13] and that *IL-6* promotes growth and survival of NB cells in the bone marrow [14]. The studies by Lagmay et al. [15] have shown that the single nucleotide polymorphism (SNP) rs1800795 in *IL-6* promoter (−174 G>C) is associated with unfavorable clinical outcome in patients affected by high-risk NB. Replication of genetic association findings remains the golden standards for results validation.

In the present study, we evaluated on a large cohort of Italian NB samples the association of the germ-line polymorphism rs1800795 of *IL-6* with NB survival. We also verified if this SNP is associated with the risk of NB initiation. Finally, we tested the SNP-gene expression association in lymphoblastoid cell lines (LCLs) and NB patients, and by using report gene assay in vitro. The influence of *IL-6* gene expression on patient survival was also evaluated.

## Materials and Methods

### Ethics Statement

This study was approved by the Ethics Committee of the Medical University of Naples and written informed consent was obtained by all children's legal guardians.

### Study population

The study consisted of 326 DNAs extracted from peripheral blood of NB patients collected through the Italian Neuroblastoma Group and 511 DNAs from cancer-free controls of Italian origin (mean age 9.67±5.41 year) (Table 1). The control subjects were recruited from Italian blood centers and were included into the study after checking for ethnicity and ancestor origins and for absence of serious underlying medical disorders, including cancer. The age of controls is significantly higher than that of cases. We selected this kind of control group to increase the power of detecting the true associations because the probability to get NB decreases with increasing age. Older control individuals are less susceptible to developing NB. Indeed, the average age at diagnosis is about 1 to 2 years, with the majority of cases presenting before the age of five years (Surveillance, Epidemiology and End Results, http://seer.cancer.gov/). Our control group consisted of 205 (40.1%) females and 306 (59.9%) males without differing from case group.

**Table 1:** Case-Control study of *IL-6*–176 (G>C) SNP.

	Cases n = 326	Controls n = 511	P	OR (CI = 95%)
**Genotype**				
GG	176 (0.54)	295 (0.58)	a0.24	a1.14
GC	125 (0.38)	184 (0.36)		
CC	25 (0.08)	32 (0.06)		
GG/GC	301 (0.92)	479 (0.94)	0.34	1.31
CC	25 (0.08)	32 (0.06)		(0.75–2.28)
**Allele**				
G	477 (0.73)	774 (0.76)	0.24	1.14
C	175 (0.27)	248 (0.24)		(0.92–1.43)

a P-value and OR obtained by Armitage's trend test.

Clinical and biologic characteristics of the patients are shown in Table 2. Samples were assigned into two risk groups (not high-risk and high-risk) based on the COG risk assignment [1]. International Neuroblastoma Staging System (INSS) stage 4, age at diagnosis greater than 18 months and amplification of the oncogene *MYCN* are the most important predictors of poor outcome [1]. Tumor specimens were collected at the onset of disease from 31 patients who were diagnosed with a primary NB between 1990 and 2006, and referred to the Gaslini Children Hospital, Genoa, Italy. The characteristics of tumors are reported in Table S1. Genomic DNA from NB and control samples was isolated using the Wizard Genomic DNA Purification Kit (Promega Corporation, Madison, WI).

**Table 2:** Characteristics of NB patients stratified per *IL-6* -176 (G>C) SNP genotype.

		Genotypic frequencies						
Variables	n	GG	GC	CC	aP	OR (CI = 95%)	bP	OR (CI = 95%)
**Age**								
≥18 months	152	77 (0.50)	63 (0.41)	12 (0.08)	0.39	1.17 (0.82–1.65)	0.65	1.22 (0.52–2.87)
<18 months	168	94 (0.56)	62 (0.37)	12 (0.07)				
N. A.	6							
**Sex**								
Male	178	92 (0.52)	72 (0.40)	14 (0.08)	0.41	1.16 (0.81–1.65)	0.52	1.34 (0.55–2.35)
Female	141	79 (0.56)	53 (0.38)	9 (0.06)				
N. A.	7							
**INSS stage**								
Stage 1+2	103	57 (0.55)	44 (0.43)	2 (0.02)	c0.21	1.29 (0.87–1.91)	c0.02	5.03 (1.13–22.46)
Stage 3+4	192	102 (0.53)	72 (0.37)	18 (0.09)				
Stage 4S	23	12 (0.52)	8 (0.35)	3 (0.13)				
N. A.	8							
**Risk**								
High-risk	144	73 (0.51)	55 (0.38)	16 (0.11)	0.08	1.36 (0.96–1.92)	0.03	2.51 (1.05–5.99)
Not high-risk	182	103 (0.57)	70 (0.38)	9 (0.05)				
**MYCN**								
Amplified	73	35 (0.48)	32 (0.44)	6 (0.08)	0.17	1.33 (0.88–2.02)	0.33	1.66 (0.59–4.69)
Not Amplified	221	126 (0.57)	82 (0.37)	13 (0.06)				
N. A.	32							

N.A.  =  not available.

a p-values and ORs from comparison of allelic frequencies.

b p-values and ORs from comparison of genotype frequencies (GG/GC vs CC).

c p-values and ORs from comparison of stage 1+2 patients vs stage 3+4 patients.

### rs1800795 (−174 G>C) genotyping

Patient's genotype was identified by DNA samples screening for the SNP rs1800795 using Restriction Fragment Length Polymorphism (RFLP) mapping strategy, as described by Lagmay et al. [15]. Briefly, the method was PCR based and used primers that flank the SNP locus to produce an amplicon of 305 bps. The PCR products were gel visualized on 2% agarose and digested with the DNA restriction endonuclease Nla-III. The DNA primer sequences used were as follows: forward-*ATGCCAAGTGCTGAGTCACTA*; reverse-*TCGAGGGCAGAATGAGCCTC*. For quality control, 10% of randomly selected samples containing both cases and controls were analyzed at second time by Sanger sequencing (3730 DNA analyzer, Applied Biosystems) without finding any discrepancies.

### *In silico* SNP-gene expression correlation analysis

The correlation between the SNP rs1800795 and *IL-6* gene expression was evaluated using the web tool SNPexp v1.2 (http://app3.titan.uio.no/biotools/tool.php?app=snpexp) [16] as already performed in our previous study [8]. SNPexp calculates correlation between HapMap genotypes and gene expression levels in LCLs using linear regression. For this analysis, 198 unrelated HapMap3 subjects were chosen.

### *In silico* analysis of *IL-6* gene expression-outcome correlation

Two independent sets of normalized gene expression data and clinical annotations (Table S2 and Table S3) were downloaded from the website R2 microarray analysis and visualization platform (http://hgserver1.amc.nl/cgi-bin/r2/main.cgi):

1. Versteeg dataset composed of 88 NB samples;
2. Seeger dataset composed of 102 NB samples.

### *IL-6* gene expression analysis in primary tumors

RNA from 31 NB tissues was extracted using the TRIzol reagent (Invitrogen Life Technologies). Two micrograms of total RNA were reverse transcribed into cDNA using iScript cDNA Synthesis Kit (Biorad). To evaluate the gene expression of *IL-6*, quantitative real time PCR (qRT-PCR) was performed using Power SYBR Green Master Mix (Applied Biosystems). Samples were amplified on an Applied Biosystems 7900HT Sequence Detection System using standard cycling conditions and data were collected and analyzed by 2?^−Δct^ method as described previously [17]. The normalization factor was calculated based on the arithmetic mean Ct value of two stably expressed reference genes (*HPRT* and *ACTB*). *HPRT* was used as housekeeping gene. Primers used are specific for the full length *IL-6* isoform and overlapped the exon-exon junction (*IL-6* forward: *TCTCCACAAGCGCCTTCGGT, IL-6* reverse: *TGGGGCAGGGAAGGCAGC; HPRT* forward:*TGACACTGGCAAAACAATGCA, HPRT* reverse: *GGTCCTTTTCACCAGCAAGCT, ACTB* forward: CGTGCTGCTGACCGAGG, *ACTB* reverse: GAAGGTCTCAAACATGATCTGGGT).

### Construction of luciferase reporter gene plasmids

PCR primers contained recognition sites for BglII in the forward primer and HINDIII in the reverse primer were designed to amplify a 1 kb fragment (−900+70) flanking the *IL-6*–174 G>C polymorphism. IL-6 was amplified from the genomic DNA of a healthy subject homozygous for the −174 C allele. After cutting the fragment with HINDIII and BglII restriction enzymes (Takara, Dalian, China) we cloned it into the pGL3-basic luciferase vector (Promega, Madison, WI, USA). The resulting plasmid containing the C alleles at nucleotide position −174 was site-specifically mutated to G alleles using Site-Directed Mutagenesis Kit (Stratagene, La Jolla, CA, USA) for creating the plasmids p-GG and p-CC. Before cell transfection, the sequence of each construct was confirmed by direct sequencing.

### Transient transfection and luciferase reporter gene assays

HEK293 cells were transfected with 1 ug pGL3-basic constructs with different *IL-6* promoter genotypes or 1 ug pGL3-basic empty plasmid (as a promoterless control) using Trasfectin (Biorad). Thirty-two nanograms Renilla pRL-TK plasmid (Promega, Madison, WI, USA) was cotransfected as a normalizing control. Forty-eight hours later luciferase activity of the transfected cells was determined using the Dual-Luciferase Reporter Assay System (Promega, Madison, WI, USA) on a TD20/20 Luminometer (Turner Designs, Sunnyvale, CA, USA). For each plasmid construct, three independent transfection experiments were carried out, and each was done in triplicate. Results are reported as relative luciferase activities, which are obtained by dividing firefly luciferase activity with Renilla luciferase activity.

### Statistical analysis

The statistical power was calculated by the software Quanto (http://hydra.usc.edu/gxe/) and StatTools (http://www.stattools.net/index.php). In case-control study, we had more than 80% power at a P value of 0.05 to detect an association with SNP alleles conferring a genotype relative risk greater than 1.3. In the survival analysis, we had more than 80% power at a P value of 0.05 to detect a 20% difference in 5-year of overall survival (OS) (60–80%). Hardy-Weinberg equilibrium was evaluated using the goodness-of-fit chi-square test in control subjects. Two-sided chi-square tests were used to test for associations of the existence of polymorphism versus each other clinical factor, all patients, and only high-risk patients. Odds ratios (ORs) and 95% confidence intervals (CIs) were calculated to assess the disease risk conferred by a specific allele or genotype. OS was calculated by Kaplan-Meier method to generate survival curves which were compared using a log-rank test. Patients of Versteeg and Seeger datasets were dichotomized into both a high-risk group and a low-risk group, using the 50th percentile (median) cutoff of the normalized gene expression data as the threshold value. The normalized gene expression and clinical data of the two datasets are reported in the Table S2 and S3. The Cox regression model was used to test for the independent predictive ability of the SNP after the adjustment for other significant factors: *MYCN* amplification, age, INSS stages. Concordance-index (C-index) was calculated using the “coxph” package implemented in R environment. *P*-values <0.05 were considered statistically significant.

## Results

### *IL-6* SNP genotyping

*IL-6* SNP rs1800795 allele frequencies for NB patients and controls are shown in Table 1. No significant association was found with NB risk when allele and genotype frequencies of the *IL-6* polymorphism were compared between patients and control individuals (Table 1). The genotype distribution was in agreement with Hardy–Weinberg equilibrium. The genotype CC was significantly more frequent than GG/GC both in high-risk patients (*p* = 0.03) and in patients at stage 3 or 4 (*p* = 0.02) (Table 2).

### OS association

**Figure 1:**
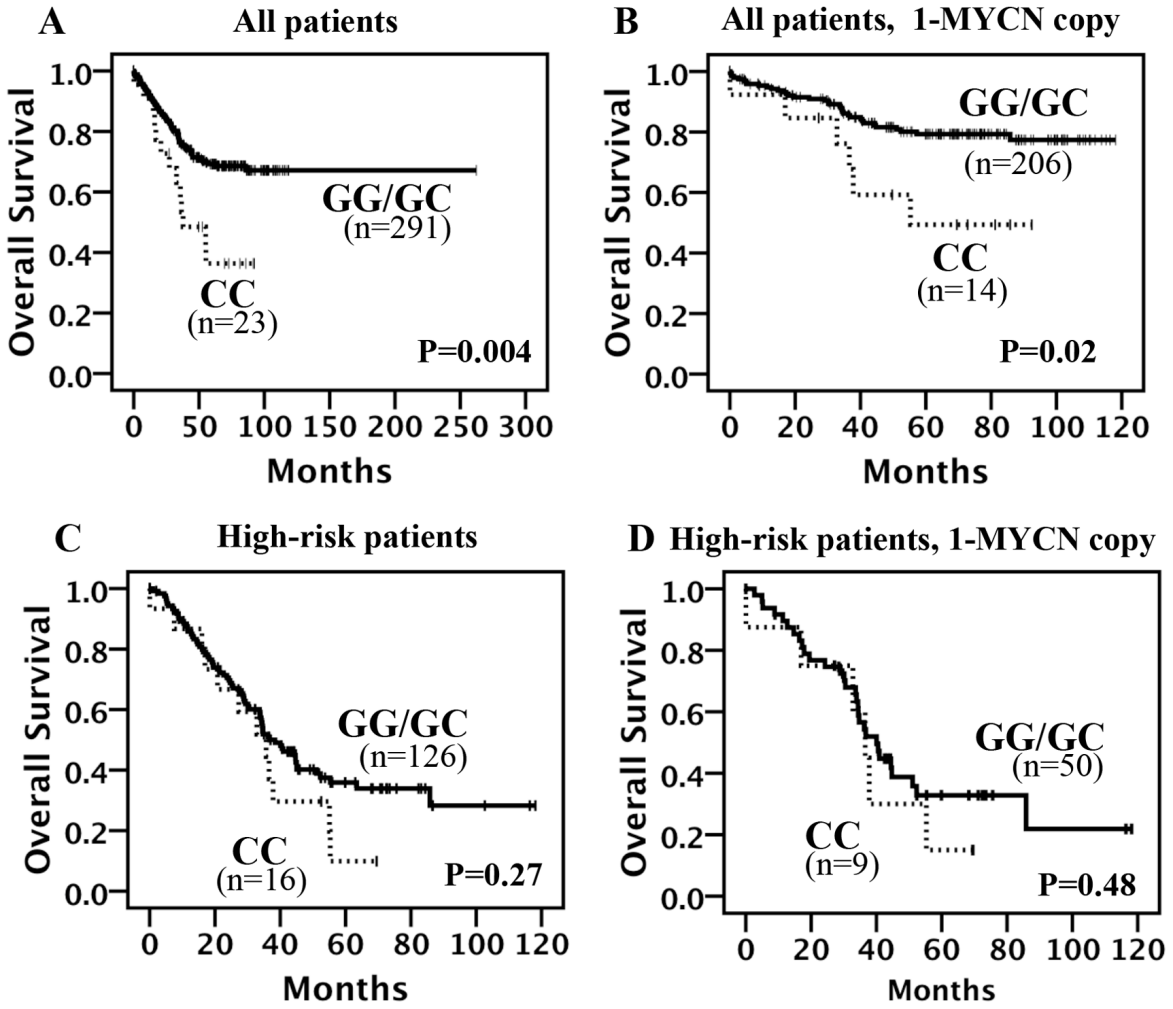
Kaplan-Meier curves for OS rates. OS rates were compared between CC and any G (GC or GG) for the SNP *IL-6*–174 in (A) all patients, (B) in not *MYCN* amplified patients, (C) in high-risk patients and (D) not *MYCN* amplified high-risk patients.

*IL-6* SNP genotypes showed statistically significant differences (*p*<0.05) in NB outcome prediction. Patients homozygous for the C allele (CC) had worse survival than patients homozygous and heterozygous for the G allele (GC and GG) (Figure 1A). In particular, OS at 5 years was 85.2% (95% CI 0.83–0.87) for the group of patients carrying GG and GC genotype while only 67.2% (95% CI 0.55–0.79) for the group of patients carrying CC genotype (Figure 1A). In the sub-group of patients with *MYCN* single copy, the genotype homozygous (CC) was still associated to worse survival (Figure 1B) suggesting that *IL-6* SNP rs1800795 association to survival is independent from *MYCN* status. A similar trend, without reaching the significance, was observed when the analysis was restricted to high-risk patients (Figure 1C and 1D). The −174 *IL-6* SNP remained independently prognostic factor after adjustment for age ≥18 months (model A), or for MYCN amplification (model B) or for INSS stage 4 (model C) (Table 3). To assess the predictive accuracy of *IL-6* SNP, we calculated the C-index. In this fashion, a C-index value of 0.5 indicates predictive performance which is no better than chance, whereas values greater than 0.5 indicate true predictive capacity. The predictive power of the clinical markers age, *MYCN*, and INSS stage improved when *IL-6* SNP was included in the model (Table 3).

**Table 3:** Test for independent statistical significance of −174 *IL-6* SNP after adjustment for NB risk factors.

Model	HR (95% CI)	P	aC-index	bC-index
A.				
Age (≥18 months vs <18 months)	4.18 (2.55–6.84)	<0.0001	0.64	0.65
−174 IL6 SNP (CC vs GC/GG)	1.90 (1.05–3.43)	0.03		
B.				
MYCN (amplified vs not amplified)	3.85 (2.48–5.98)	<0.0001	0.72	0.73
−174 IL6 SNP (CC vs GC/GG)	1.95 (1.03–3.68)	0.04		
C.				
INSS Stage (4 vs 1–2–3–4s)	9.06 (5.11–16.08)	<0.0001	0.64	0.66
−174 IL6 SNP (CC vs GC/GG)	1.90 (1.05–3.42)	0.03		

HR, hazard ratio; CI, confidence interval; C-index, concordance index.

a C-index calculated including in the model only the NB risk factor (Age or MYCN or INSS stage 4).

b C-index calculated including in the model the NB risk factor and *IL-6* SNP.

### *IL-6* SNP rs1800795 gene expression analysis and report gene assay

**Figure 2:**
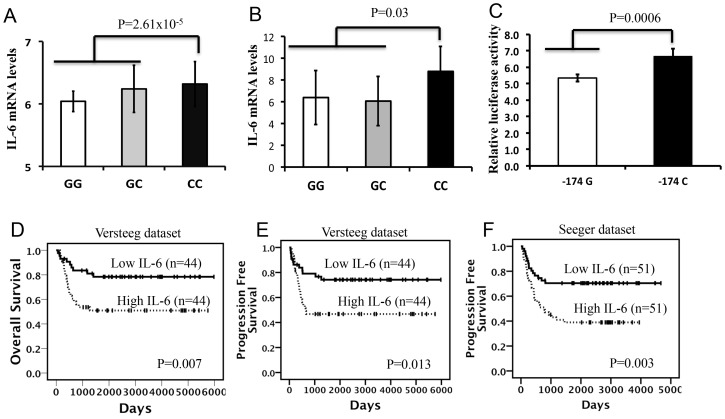
SNP genotype correlation to *IL-6* gene expression and NB outcome. *In silico* and qRT-PCR analysis of *IL-6* mRNA expression in (A) 198 LCLs and (B) 31 NB tumors, respectively, stratified according to the SNP *IL-6*–174. (C) Luciferase report gene assay carried out in HEK293 cells. Data shown in percentage are the mean ± SD from three independent transfection experiments, each done in triplicate and compared with promoter less control. Kaplan-Meier analysis is shown, with individuals grouped by median of expression of *IL-6* for OS and Progression Free Survival (PFS) rates in (D and E) 88 NB patients and (F) only PFS for 102 INSS stage 4 patients with *MYCN* not amplified. OS data of Seeger dataset were not available.

*In silico* analysis showed that the SNP CC genotype correlated with high level of *IL-6* expression in LCLs (*p* = 2.61×10^−5^, Figure 2A). We observed the same correlation by performing qRT-PCR on NB tumor specimens. We found that *IL-6* mRNA levels were higher in CC carriers than in GG and GC carriers (*p* = 0.03, Figure 2B). Furthermore, the induction promoter activity of the construct containing −174 C was higher than that of the construct containing −174 G (*p* = 0.0006) as assessed by luciferase report gene assay (Figure 2C).

### *IL-6* gene expression correlation to outcome

We verified the association of *IL-6* gene expression with disease outcome in two independent sets of NB patients; we found that the increase of the mRNA expression was significantly associated with lower OS and progression of disease (Figure 2D, 2E, and 2F).

## Discussion

*IL-6* is crucial cytokine involved in several cellular pathways [9]. This article underlies the role of *IL-6* as marker of NB progression. This hypothesis is supported by scientific findings showing that *IL-6* has a protective effect on drug-induced apoptosis treatment in NB cells [14] and that its expression as well the expression of *IL-6* receptor is inhibited upon treatment with retinoic acid, currently used agent in NB therapy [18]. Peripheral blood and bone marrow *IL-6* levels have been found to be elevated in patients with high-risk NB when compared with those with low and intermediate risk NB [13].

In previous studies, SNP −174 G>C in *IL-6* promoter has been associated to diverse tumors but the reported results are contradictory regarding the genotype associated to tumor progression [19]–[26]. A recent study by Lagmay et al. [15] shows the genotype GG of the *IL-6* SNP −174 G>C as responsible of a worse outcome in patients with high-risk NB. Here we performed a genetic study of *IL-6* SNP −174 G>C in an Italian cohort of NB specimens by analyzing a greater number of affected children in addition to a large sample of healthy individuals with respect to previous study [15]. Our data suggest that this SNP does not predispose to NB development but is associated with NB progression. We found that overall Italian NB patients homozygous for the C allele had a worse outcome than patients homozygous or heterozygous for the G allele. The restricted analysis to high-risk NB patients resembles the same association trend without reaching significant threshold. Our results concerning the genetic association between *IL-6* CC genotype and high-risk phenotype, advanced INSS stage, and the effect of SNP *IL-6*–174 on survival in NB specimens are contradictory to that of Lagmay et al. [15]. Our study was planned to detect the differences in *IL-6* SNP frequency in an Italian NB population. We calculated the sufficient number of samples by power analysis to detect genetic risk for NB development and progression based on *IL-6* SNP frequency. This sample was assessed to achieve more than 80% power at P value of 0.05. Moreover, our findings are strengthen by the fact that the same Italian NB cohort has been successfully used to validate GWAS-identified NB risk variants [4]–[6], [8]. We designed a robust replication study that unexpectedly has shown an opposite *IL-6* SNP association with NB progression respect to that obtained in Lagmay study [15]. This points out the need for performing additional replication studies to confirm our observation.

In order to associate the genotype to gene expression, we also evaluated the impact of SNP *IL-6* –174 on *IL-6* gene expression in LCLs and NB specimens. We used both models (LCLs and NB tissues) since the large percentage of pauciclonality in LCLs combined with widespread monoallelic expression profiles has been shown to lead to differential expression profiles between LCLs and *ex vivo* cells [27]–[28]. On the other hand, diverse studies have compared the overlap of expression Quantitative Trait Loci (eQTLs) found in LCLs and primary tissues and found that a large number of eQTLs detected in LCLs can also be detected in primary tissues [29]–[31]. In previous works it has been found that C allele was associated with increased *IL-6* expression levels [20]–[22], whereas in other studies the C allele was associated with lower *IL-6* expression levels [19], [24]. In our study we observed an increased of *IL-6* mRNA expression in C allele homozygous subjects. This result was further confirmed by using a report gene assay. The contradictory reports regarding correlation of *IL-6*–174 G>C SNP and *IL-6* expression may partially be explained by nearby SNPs (−597, −572 and −373) that also affect the activity of the *IL-6* promoter [32]. Furthermore, *IL-6* seems to be regulated differently depending on the type of cell it is expressed in [32]. We are pretty confident in our results as we found a significant association between *IL-6*–174 CC genotype and *IL-6* expression in LCLs and more importantly in NB tissues, and by luciferase assay *in vitro*. Moreover, we observed a strong association between high levels of *IL-6* and poor outcome in other two independent gene expression array datasets composed of NB patients.

Stromal-derived *IL-6* promotes osteoclast activation, the formation of osteolytic bone metastasis [33], and the resistance of tumor cells to cytotoxic drugs [14]. It has been shown that NB patients with elevated *IL-6* have a poor outcome. The consequent elevated production of *IL-6* in individuals carrying the CC genotype SNP in the *IL-6* promoter region may be a plausible explanation for these data, suggesting that *IL-6* may be a factor involved in NB disease progression. Based on previous and our findings, we speculate that *IL-6*–174 CC genotype can predispose to progression of NB disease.

As already demonstrated concerning other gene polymorphisms [1]–[8], we found that a genetic variant of *IL-6* might influence NB clinical outcome. However, additional investigations are needed to confirm the genetic association of the SNP *IL-6*–174 with clinical outcome in NB patients.

## Supporting Information

Table S1 **Characteristics of NBL tumors.**
(XLSX)

Table S2 **Gene expression and clinical data of Versteeg dataset.**
(XLSX)

Table S3 **Gene expression and clinical data of Seeger dataset.**
(XLSX)
